# Supplementary figures and images for: Charting the spatial dynamics of early SARS-CoV-2 transmission in Washington state
Source: PLoS Comput Biol. 2023 Jun 28;19(6):e1011263. doi: 10.1371/journal.pcbi.1011263 (PMC10335681; doi:10.1371/journal.pcbi.1011263)

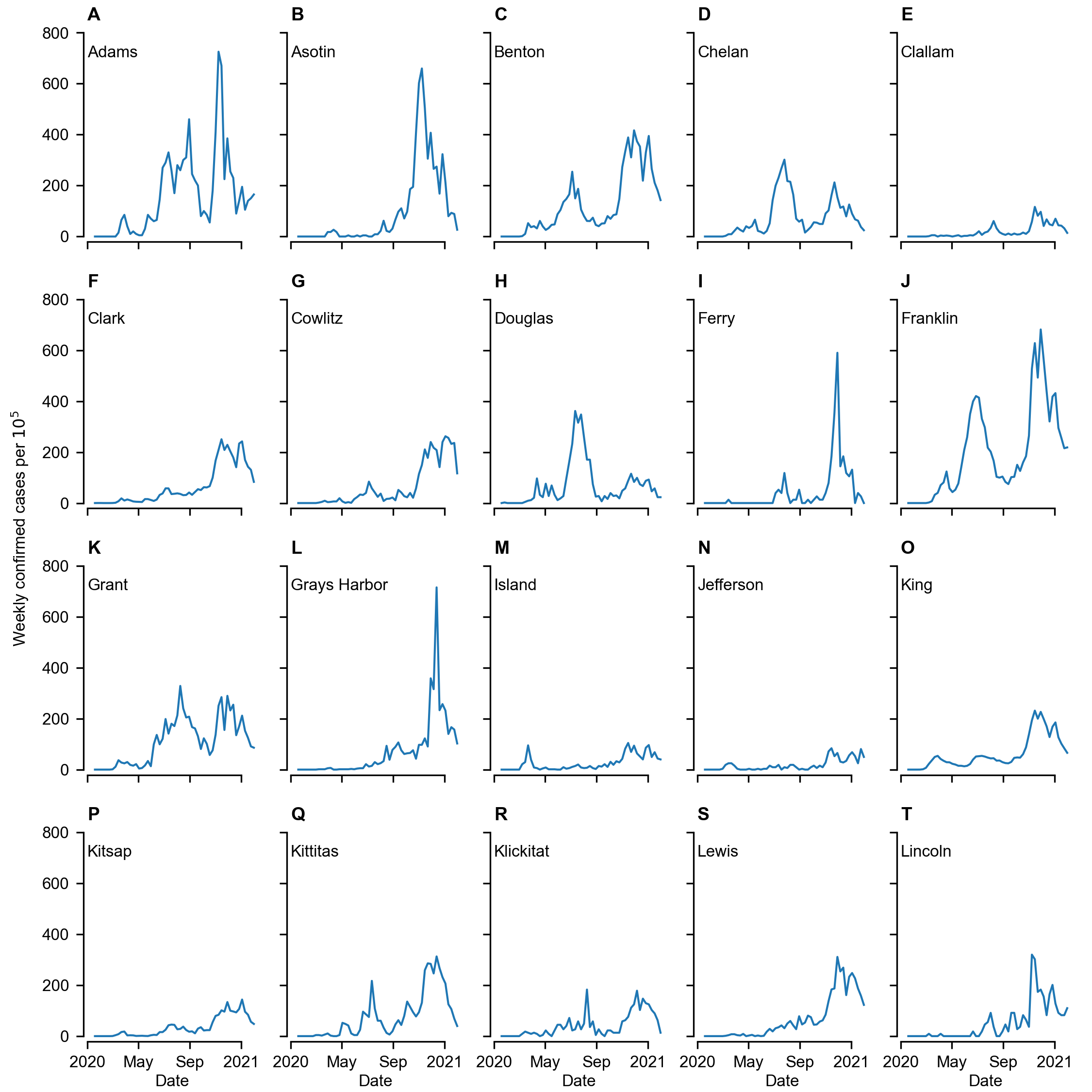

Supplement: S1 Fig — (TIFF) [file pcbi.1011263.s001.tiff]

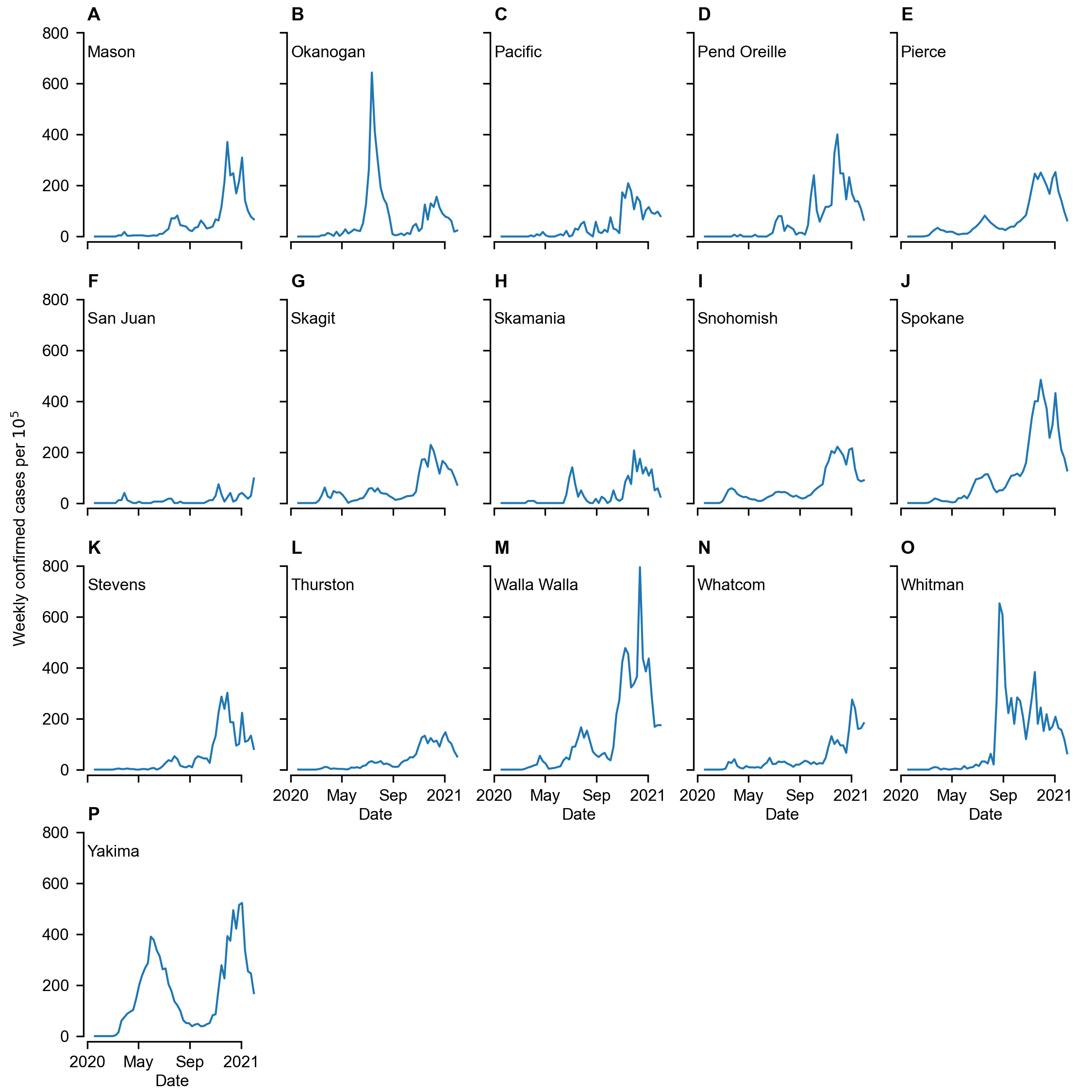

Supplement: S2 Fig — (TIFF) [file pcbi.1011263.s002.tiff]

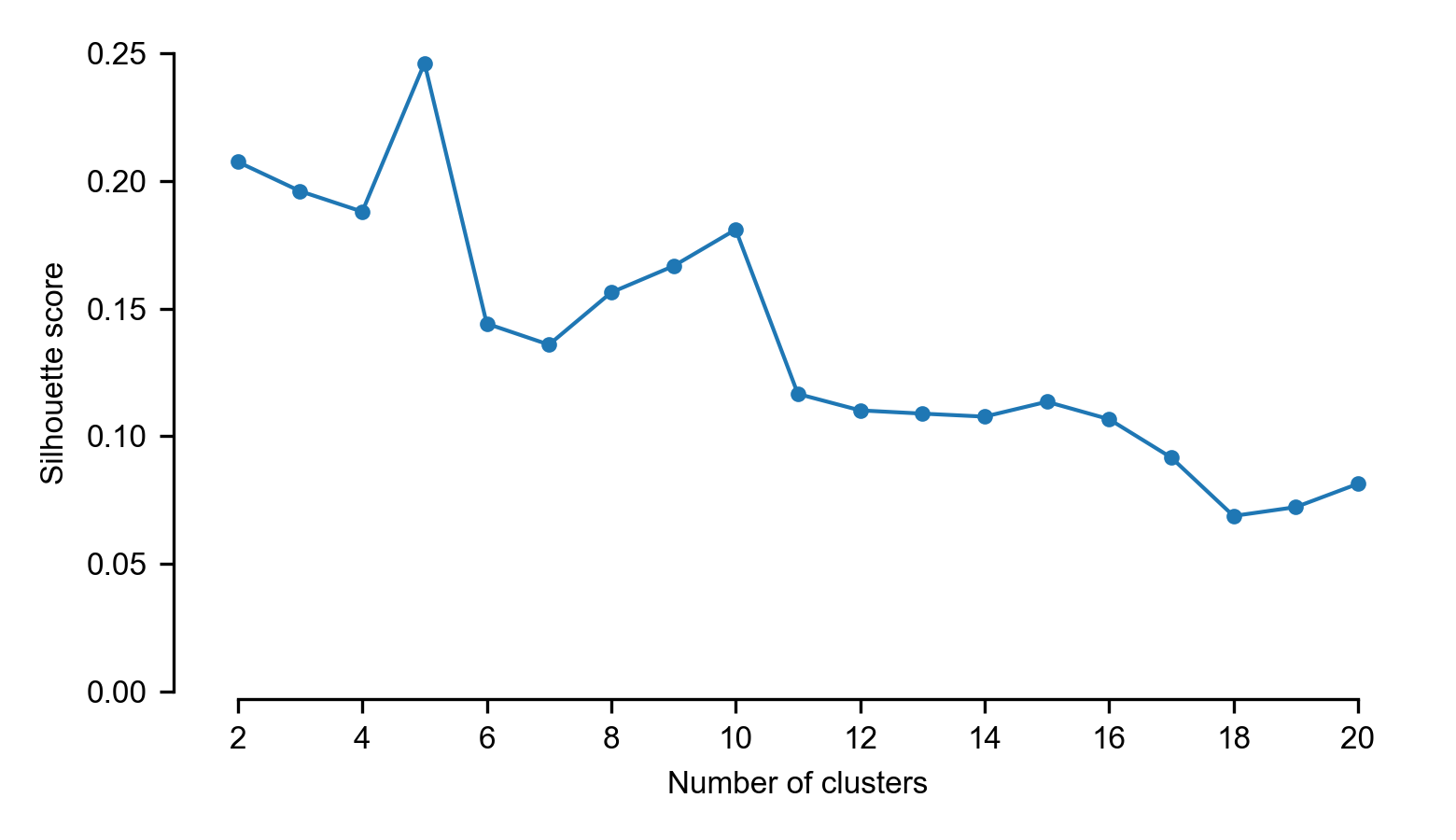

Supplement: S3 Fig — (TIFF) [file pcbi.1011263.s003.tiff]

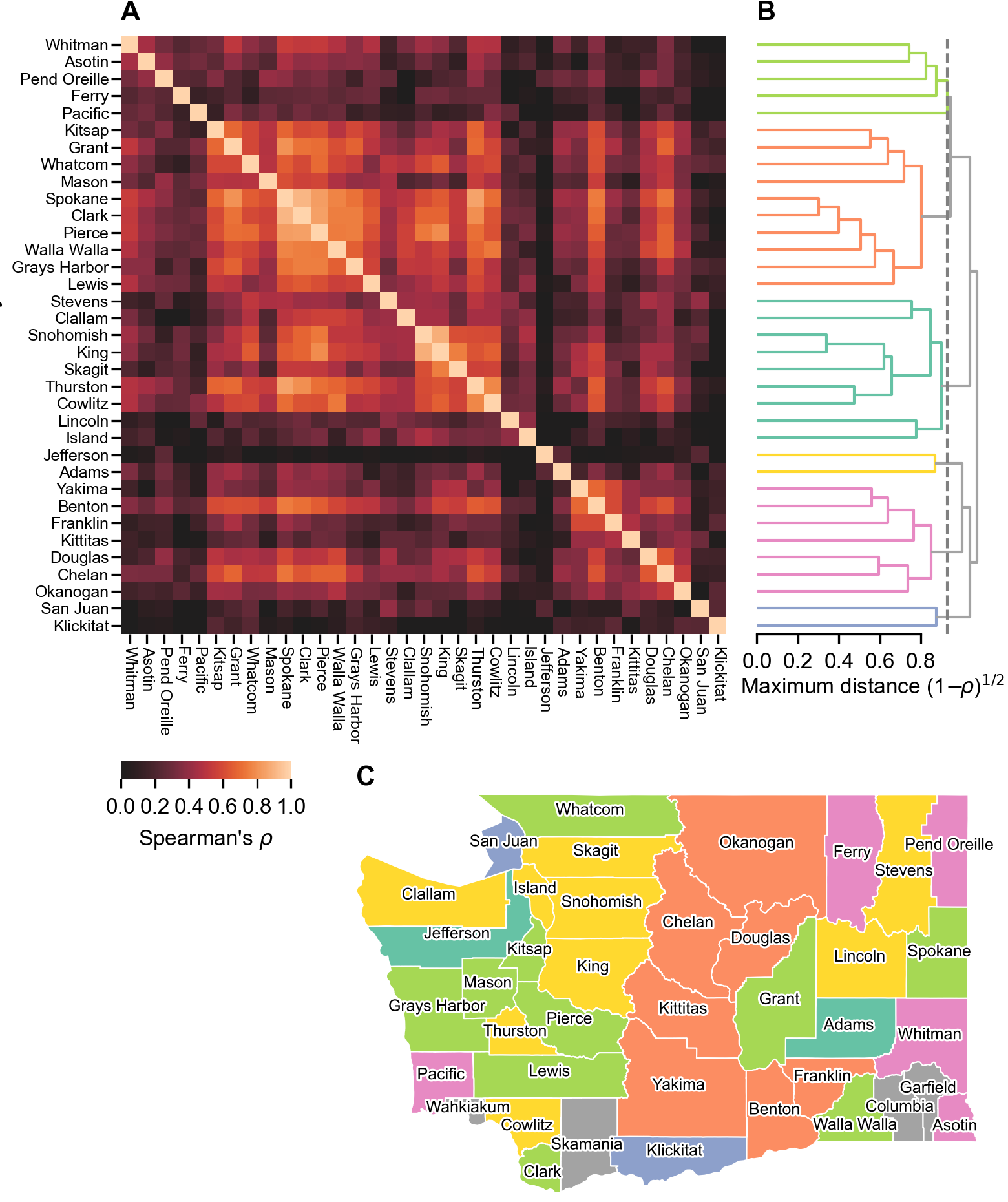

Supplement: S4 Fig — Maps makes use of TIGER/Line Shapefiles from the U.S. Census Bureau which are in the public doman (https://www.census.gov/geographies/mapping-files.html). (TIFF) [file pcbi.1011263.s004.tiff]

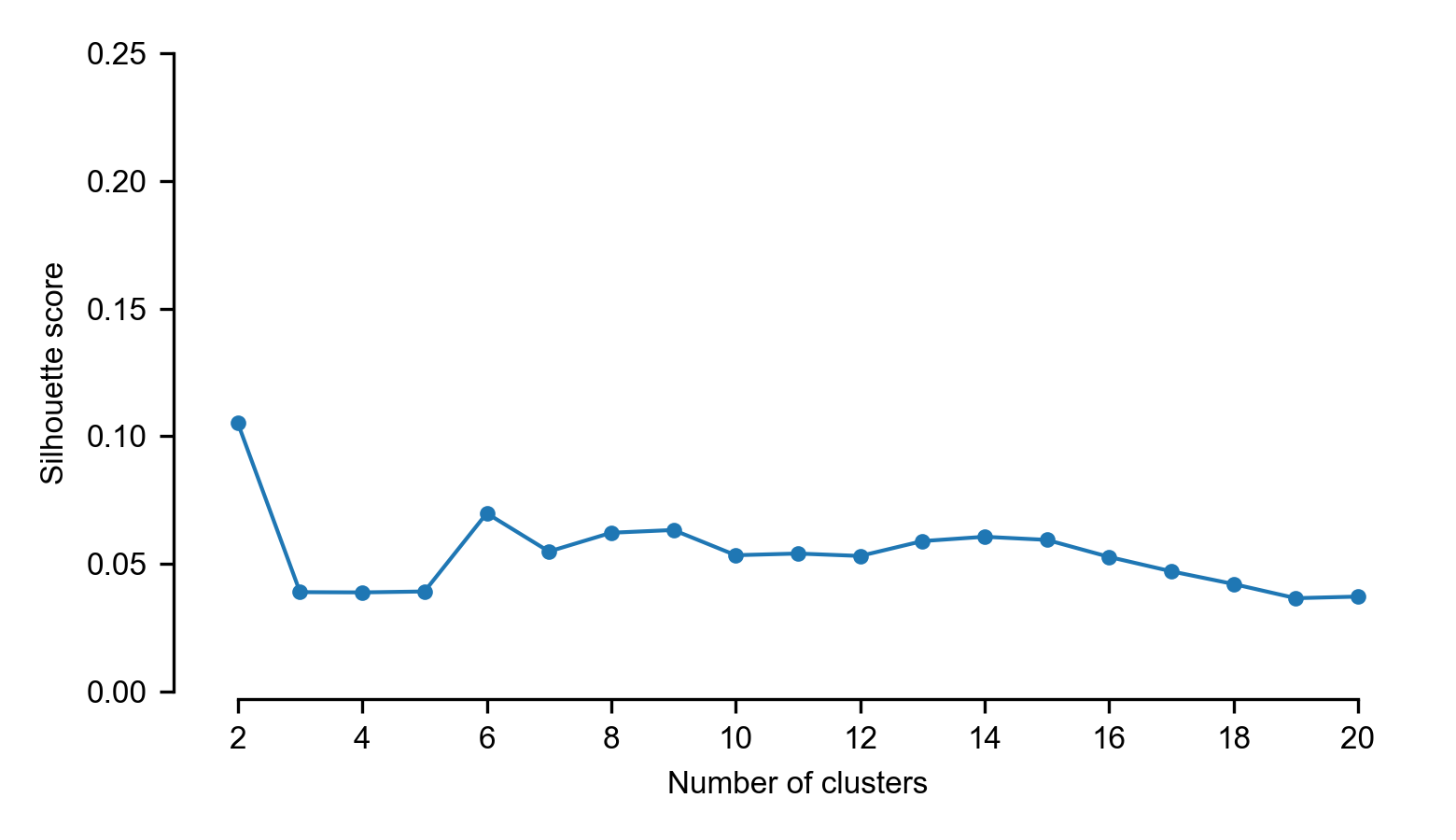

Supplement: S5 Fig — (TIFF) [file pcbi.1011263.s005.tiff]

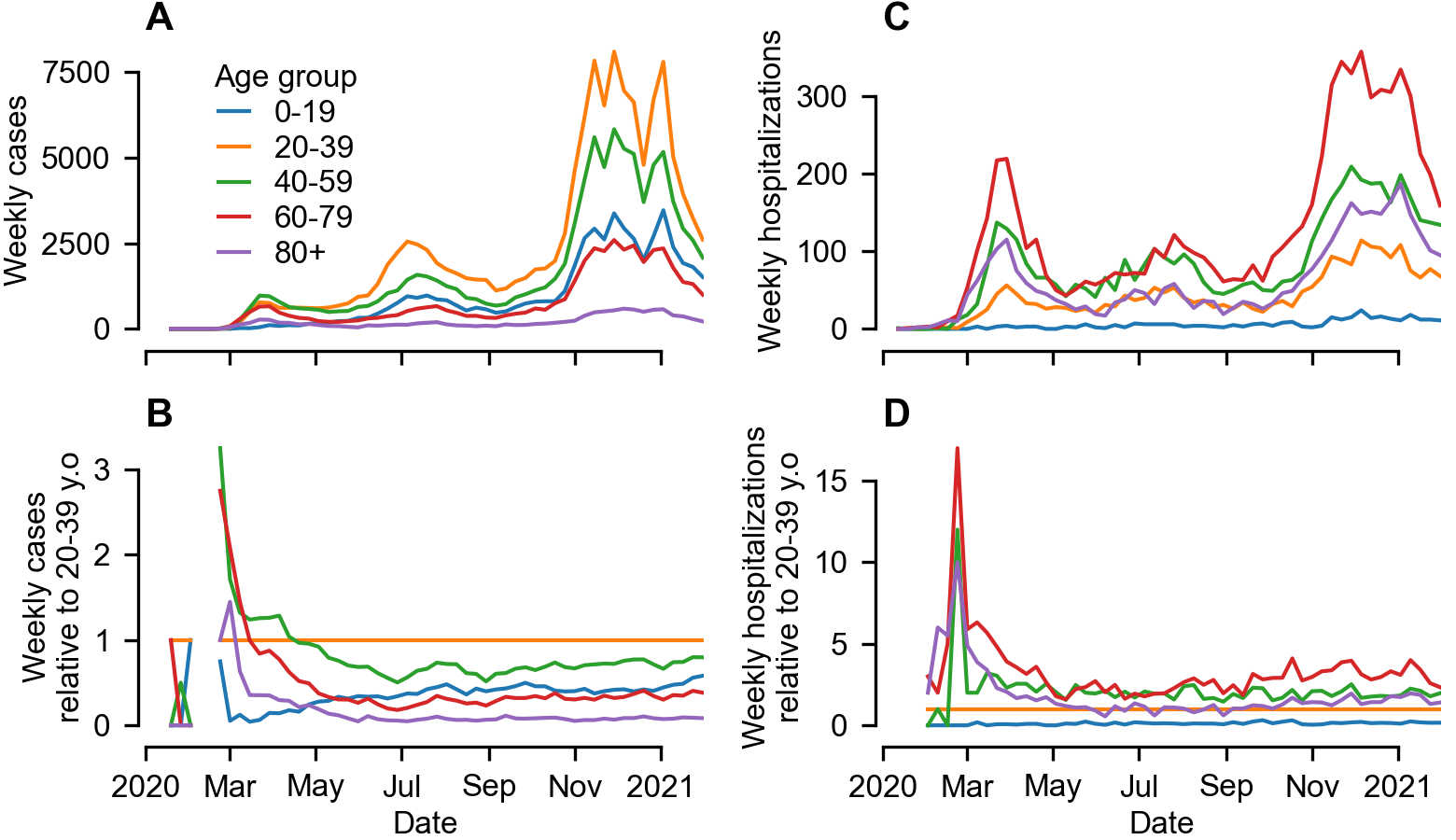

Supplement: S6 Fig — A) Weekly confirmed cases by age group B) Weekly age-specific confirmed cases relative to cases aged 20–39 y.o. C) Weekly new hospitalisations by age group D) Weekly age-specific new hospitalizations relative to hospitalizations aged 20–39 y.o. (TIFF) [file pcbi.1011263.s006.tiff]

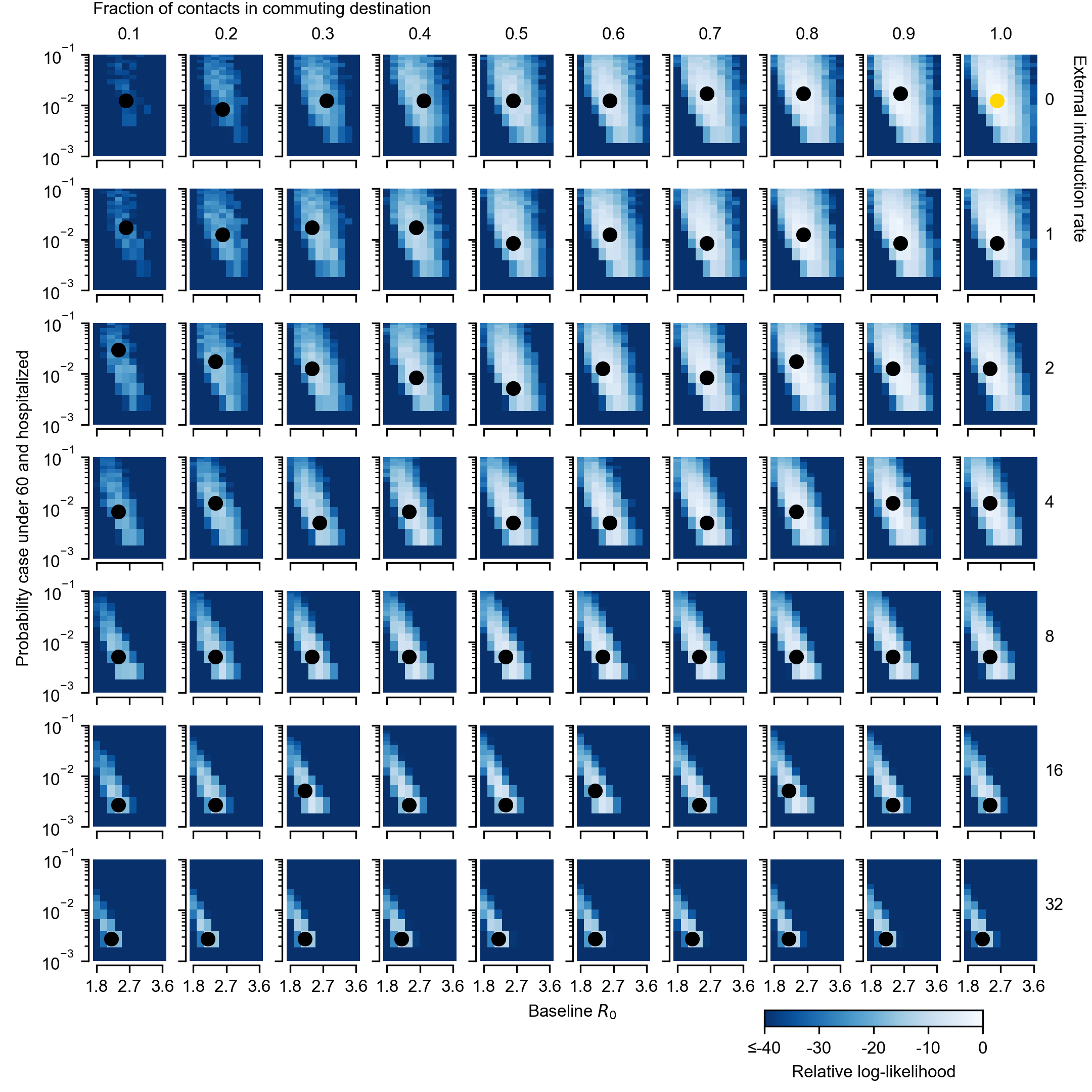

Supplement: S7 Fig — The local maximum in each panel is indicated by a black dot, with the global maximum highlighted in yellow. (TIFF) [file pcbi.1011263.s007.tiff]

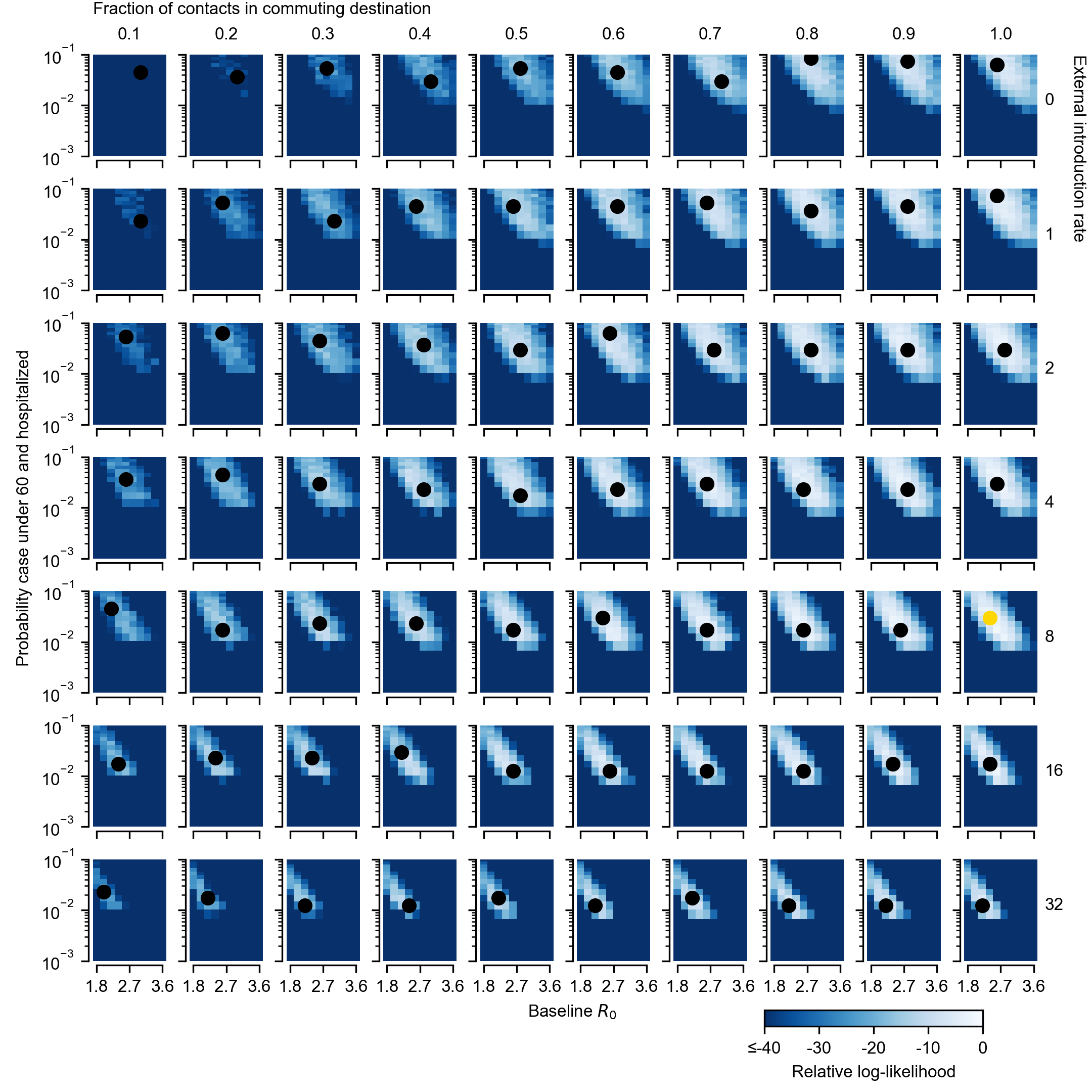

Supplement: S8 Fig — Instead of initialising the outbreak with one infection in Snohomish county in the week beginning January 12, the outbreak simulations were initialised with one infection in King county in the week of January 26 2020. The local maximum in each panel is indicated by a black dot, with the global maximum highlighted in yellow. (TIFF) [file pcbi.1011263.s008.tiff]

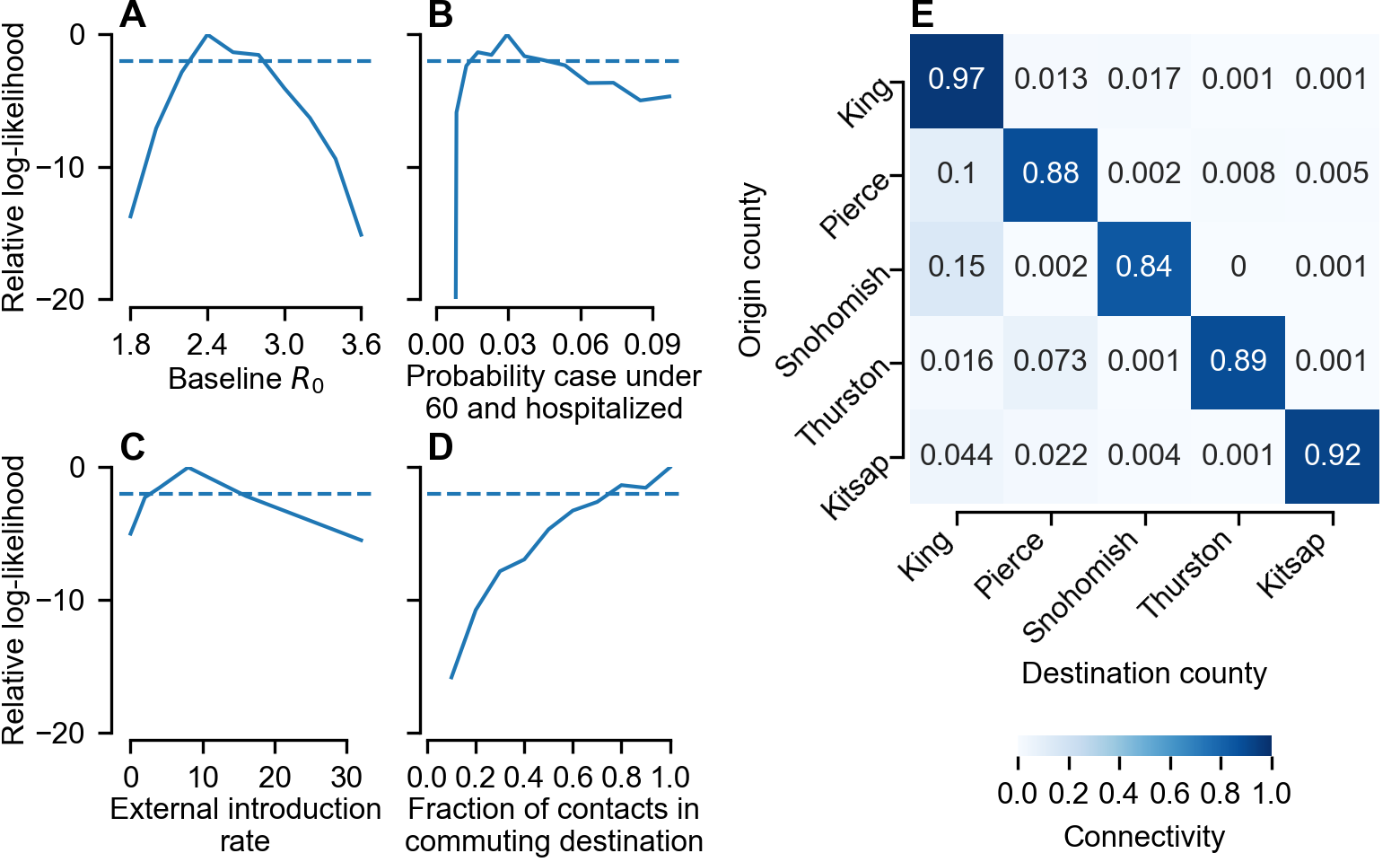

Supplement: S9 Fig — (TIFF) [file pcbi.1011263.s009.tiff]

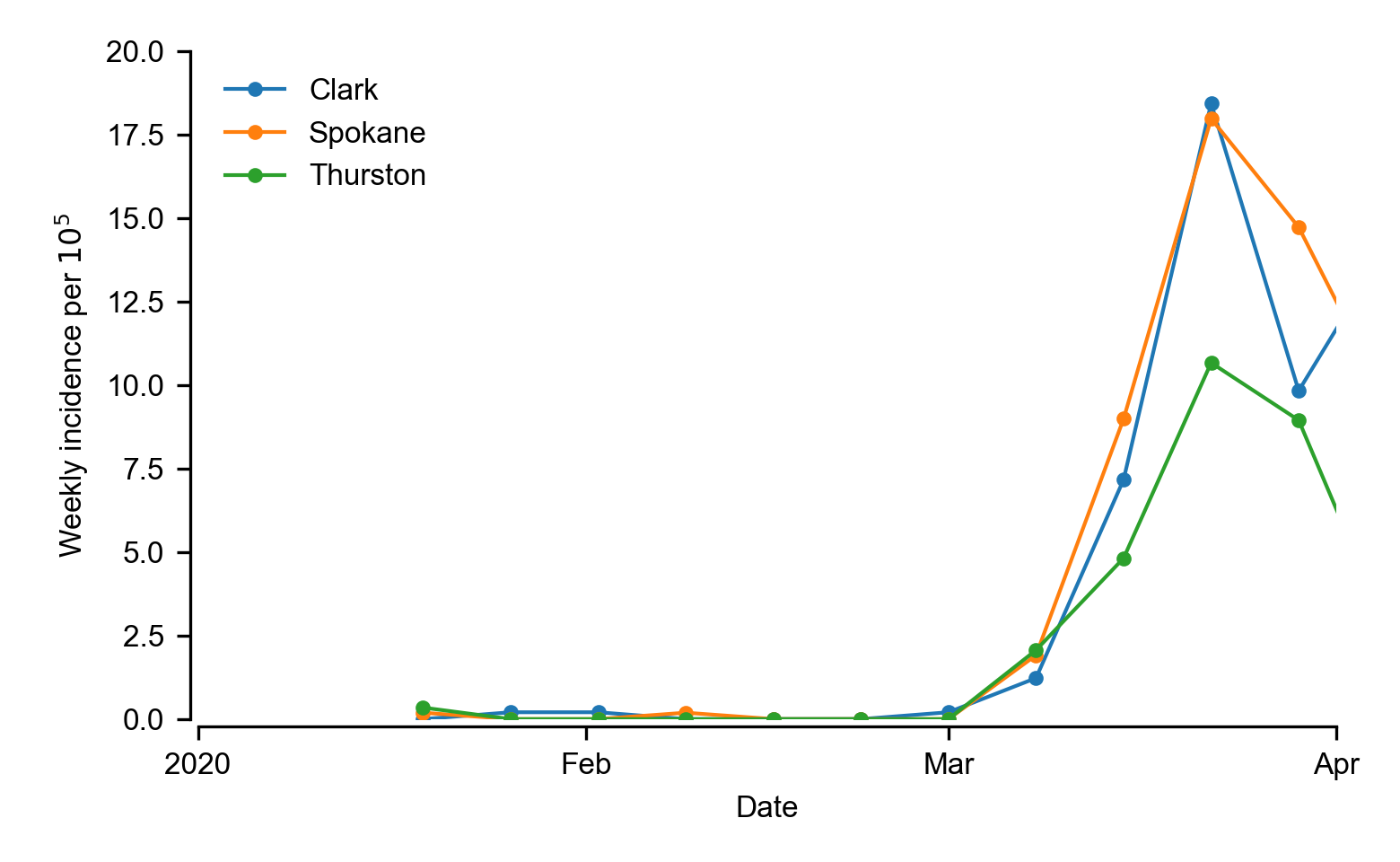

Supplement: S10 Fig — (TIFF) [file pcbi.1011263.s010.tiff]

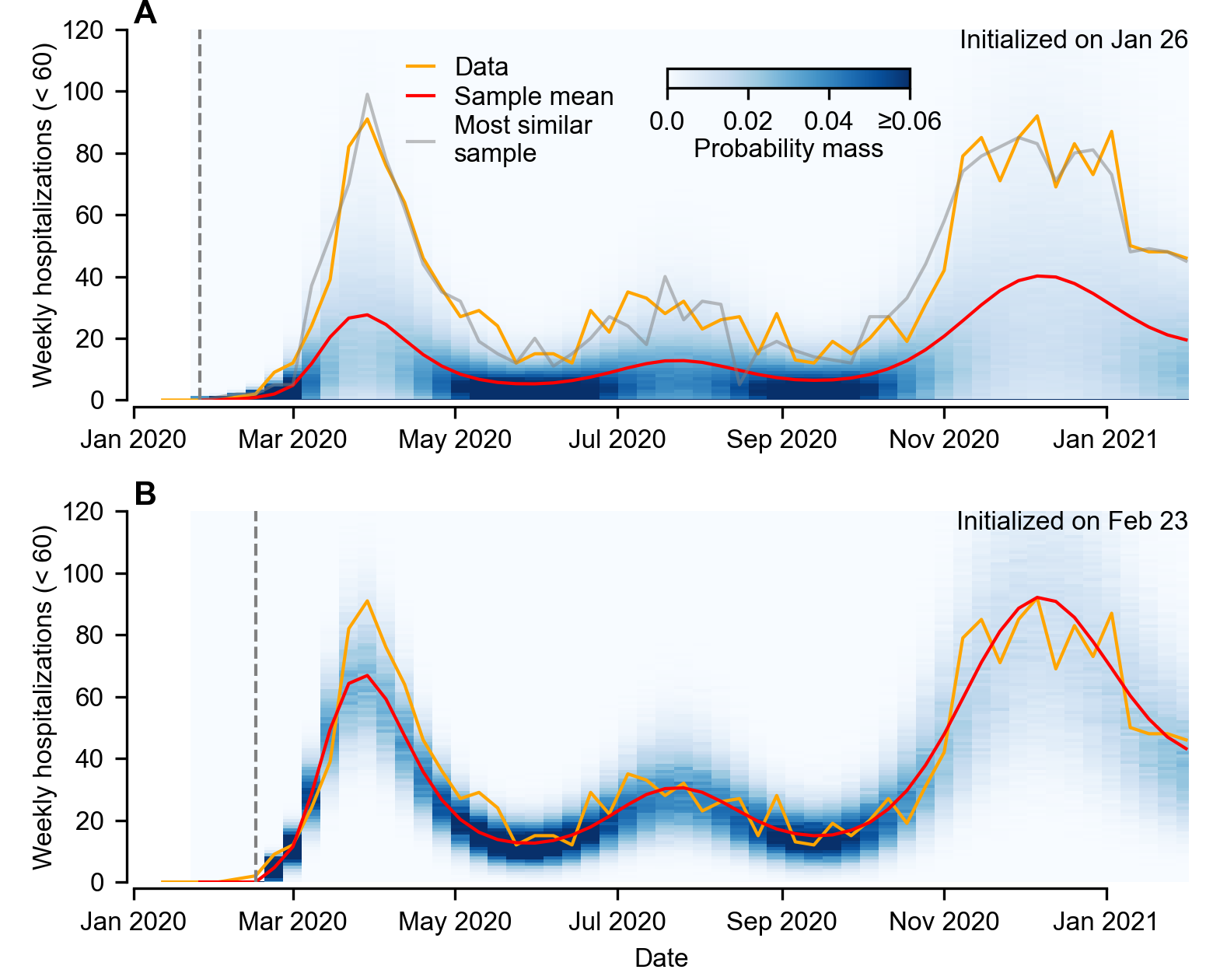

Supplement: S11 Fig — As with the results shown in S8 and S9 Figs, the outbreak was initialised with one infection in King county in the week of January 26 2020. Parameters used were those that maximised the log-likelihood (shown in S8 Fig) subject to η = 0, i.e. ensuring that the outbreak was seeded from a single introduction. (TIFF) [file pcbi.1011263.s011.tiff]
