# Supplementary material for: Charting the spatial dynamics of early SARS-CoV-2 transmission in Washington state
Source: PLoS Comput Biol. 2023 Jun 28;19(6):e1011263. doi: 10.1371/journal.pcbi.1011263 (PMC10335681; doi:10.1371/journal.pcbi.1011263)
Supplement: Attachment — Submitted filename: COVID_19_clustering_response_to_reviewers_2023_03_12.pdf [file pcbi.1011263.s012.pdf]

Response to reviewers' comments

We thank the reviewers for their careful and thoughtful comments on our manuscript. In response, we have worked hard to address them fully, including a complete re-fit of our model. Below we provide our point-by-point responses to the specific concerns/comments raised by reviewers.

Reviewer #1: The manuscript by Brett et al. describes a model for SARS-CoV-2 transmission fitted to data from Washington State. Some of the major conclusions drawn are about the role of stochasticity and mobility at different phases of the epidemic there.

Overall, the model was nicely done and its fit to data was good. Technically speaking, this is no small feat. With respect to the results and conclusions drawn, I think the authors more or less did what they could but that the insights gained, combined with the rigor of those insights, were more modest. There are a few respects described below in which the structure of the model was a limitation in how convincingly the authors could draw a certain conclusion. The impact of this work would be greater if more flexible or perhaps multiple models were used. There is also the issue that these results pertain only to one state and that none of the results are particularly surprising at this stage in the pandemic, given all the modeling work that has been published.

We appreciate that a vast volume of research on the transmission of SARS-CoV-2 has been produced since 2020. We hope our response is able to convince the reviewer of our study's rigour, the scope of results and why we view it as a worthwhile contribution to PLoS Computational Biology.

l 20 - It's a bit unconventional to refer to figures from the paper that present data in the introduction.

We viewed the introductory figure as important to introduce some of the issues surrounding the transmission of SARS-CoV-2, in particular the geographical variation in population density and the county-level variation in the epidemic trajectories. We note that only the raw data are shown, rather than the results of any analysis. We have used this presentational approach in past papers, and have not had issues from journals, including PLoS Computational Biology.

l 52 - Is there anything else this pattern could be "consistent with"? For example, the timing of case reporting.

Our analyses are based on hospitalisations data, grouped by hospital admission date rather than time of reporting. All hospitalisations added retrospectively were tabulated by Washington Department of Public Health based on their time of hospital admission. Since the data were downloaded over a year after April 2021, we believe timing of case reporting is not a factor.

As we state, our findings stem from the magnitude and spatial extent of the outbreak in Washington state at the start of April. Rolling back the clock, we find that unusually rapid spread in the first couple of weeks after introduction is the most consistent explanation for the data. The only other conceivable explanation is a number of further unobserved early (late January/early February) importations. However, given the spatial profile of the early epidemic, these importations would have had to be clustered geographically around the first identified case, rather than distributed according to the population of the Puget sound region (which is more plausible). Furthermore, our finding that a single introduction sparked the outbreak is consistent with the findings of two analyses of genetic sequence data, Refs. 7 and 8.

l 55 - This seems like a higher estimate of the probability of detecting infections than I have generally seen, especially for early in the pandemic.

We thank the reviewer for raising this point, and we have revised the manuscript to present our estimate in context with others from the literature. Figure 7 has been substantially revised to present our reconstruction of the cumulative prevalence of SARS-CoV-2 infection in the Puget sound region with four others from the literature, finding that “our simulations are broadly consistent with two studies (Davis et al. and Wu et al.), and are smaller than the estimates of Pei et al. and Reese et al. by a factor about 1.5 and 3 respectively.”

We have also added text to the discussion,

> “Our reconstruction of the cumulative prevalence of infection in the Puget Sound region suggests that over the course of 2020 about 7% of the population were exposed to SARS-CoV-2. While our reconstructed prevalence is consistent with two previous studies [38, 41], it also broadly tracks the estimated seroprevalence in Washington state published by the CDC [42]. Given this estimate has not been corrected for seroreversion [43], it likely provides an underestimate of the overall percentage of the population exposed to SARS-CoV-2. Our reconstructed prevalence is also lower that in two other studies [39, 40]. This difference could in part be explained by our use of the MLE for model parameters in our reconstruction. In fitting the data, there is a trade-off in model performance between outbreak size and the parameter ρ, the probability an infection was aged under 60 and hospitalized: As ρ decreases a larger epidemic is needed to fit the data. The 95% confidence interval on ρ allows for epidemics that are about February 21, 2023 50% larger, which would be broadly consistent with Pei et al., however the estimate of Reese et al., from a study using serological data, is

about 300% larger [40]. This discrepancy may stem from their estimate being for the combined states of Alaska, Idaho, Oregon and Washington.”

l 319 - The analysis focused on data on hospitalizations to ameliorate changes over time in reporting probability. At the same time, there was nothing in the model to help deal with the age of cases, which changed over the course of the pandemic and affected the ratio of hospitalized cases to infections.

The reviewer is correct to highlight that our modelling excluded an explicit treatment of age, and instead assumed that the age profile of infections was approximately constant over the course of 2020 (i.e. before the impact of vaccination and variants). This decision was made for computational reasons to reduce model complexity and was informed by a preliminary study of age-structured hospitalisation data, which we have now included as a supplementary figure (S6 Fig).

Broadly, this figure shows that after an initial transient period the age distribution of hospitalisations became roughly stable (panels B and D). The longer transients in the age distribution of cases (panels A and C) likely stem from changes in age specific reporting probability.

There is a lack of evidence regarding how healthcare seeking behaviour or disease severity changed through 2020 (i.e. before the wide spread of variants of concern). We therefore suspect that the transients in hospitalised cases (which primarily affect age groups over 60) are more indicative of a change in the age profile of infections (likely due to more extensive social distancing by older individuals). For this reason, we performed our inference on hospitalised cases aged under 60 y. o.. As over 60s are a comparatively small proportion of overall cases (around 15%; see panel B after the impact of transients decay), we don’t expect these trends in the age profile of infections to substantially impact our estimates of the ratio of hospitalised cases to infections.

We recognise that this is a model assumption, and have added a comment to the discussion address this, and how it might impact on some of our findings.

l 325 - The model relies on pre-pandemic mobility data from commuting patterns. Given the centrality of mobility to the results and the drastic changes that occurred to mobility early in the pandemic, this is a major concern about the study.

We used census commuting data due to a lack of publicly available data sets capturing changes in inter-county mobility. For instance, we have access to a mobility data set provided by Google which shows clear decreasing trends in metrics such as the activity at transit stations and workplaces during March and April 2020, followed by a gradual increase. Crucially, these data are presented on the county level and do not specify where individuals are travelling from/to. Since the majority of commuting is within county, trends in inter-county mobility are masked. Due to this ambiguity, we decided not to use this dataset in our final analysis.

While we do not parameterise our model with time varying mobility data, our model construction does not assume that commuters are unaffected by social distancing or that commuting rates are constant. Instead, we assume that the reduction in contact rates and mobility of commuters are determined by changes in the local reproductive number in the commuters' destination county.

We believe this is a reasonable assumption given the data available. We do not expect our estimates of the global parameters to be substantially affected as they are mostly constrained by the data prior to the introduction of social distancing. However, if inter-county mobility is more/less impacted by social distancing measures than intra-county mobility it may alter some of our estimates of the time-varying local reproductive numbers.

We gained insight into the impact of our mobility assumptions through our analysis of model performance over the course of 2020 (see Figure 5A to C). By calculating the conditional log-likelihood for each week of data and value of the commuting parameter ε, we see which weeks are fit better by different values of ε. We find that smaller values of ε had a larger conditional log-likelihood during April–June, which was the period of most intensive social distancing (Figure 5C). In the discussion we comment on this finding and its implications for modeling mobility:

> "[W]hile for most of 2020 we observed that model performance was positively correlated with connectivity strength, during the period of most intensive social distancing (April – June), this correlation was inverted. Instead, performance was better at smaller values of the commuting parameter. If non-pharmaceutical interventions resulted in a disproportionately large reduction in inter-county transmission compared to intra-county transmission, as was likely the case, this would explain the reversal in model performance. This might also explain the large drops in R_i^{res} observed in Kitsap and Thurston counties during this period, the two counties with the lowest per capita hospitalizations. Parameterising models using accurate time-varying data on human mobility would help alleviate this issue. Overall, however, our results show this second phenomenon was of smaller importance to model performance than the early rapid transmission outlined above."

methods - The model and inference procedure are sound and well-done.

We are pleased to read the reviewer's positive opinion of our approach.

methods - However, it is a bit disappointing that only 5 counties can be handled at once, presumably due to the dimensionality of the parameter space. It would be helpful if the authors could state that explicitly if so.

The issue was indeed due to the dimensionality the parameter space (which increases by $n_a = 8$ with each additional county). We have added text to the Methods clarifying this,

> “As each county had distinct transmission parameters (see below), the the curse of dimensionality limited the computational feasibility of our statistical inference to $n_c = 5$. We chose to focus on the five counties which form the core of the Puget Sound region: King, Pierce, Snohomish, Thurston and Kitsap.”

methods - I also did not see how uncertainty on model parameters was quantified given the maximum-likelihood approach.

Thank you for this point. This is an oversight on our part— we used a likelihood-ratio test. We have added text to the methods to clarify this,

> “Confidence intervals on the global parameters were constructed in a similar way: For each value of the parameter of interest, the profile likelihood was calculated by maximising the likelihood surface over all the other grid dimensions. Statistical significance was then quantified using a likelihood-ratio test and the application of Wilks’ theorem, with results shown at the 95\% confidence level.”

l 130 - Modify "above" to "in the methods" since the methods is located at the end of the manuscript.

We understand where the reviewer is coming from, but two paragraphs prior to this we state that "we introduced for each county a resident reproductive number, R_i^{res}, defined as the expected number of cases caused by a non-commuting individual who remains in county i in a fully susceptible population.” The use of “above” on line 130 is in reference to this.

l 166 - While the atuhors' investigation of what drove the estimate of epsilon is useful, it eschews the larger issue of the fact that mobility varied substantially during this period. While they account for that with time varying reproduction number, it certainly should have resulted in a time varying epislon, too. This highlights a serious constraint of the model in that it fixes epsilon over time.

As the reviewer mentions, our model assumes that reduction in the mobility of a commuter is determined by changes in the local reproductive number in the commuter’s destination county. We have modified the text to make clear that epsilon is the "baseline pre-social distancing fraction of total contacts a commuting individual would have in their commuting destination.” We do not vary the strength of commuting beyond this due to a lack of mobility data to constrain it (see our response above).

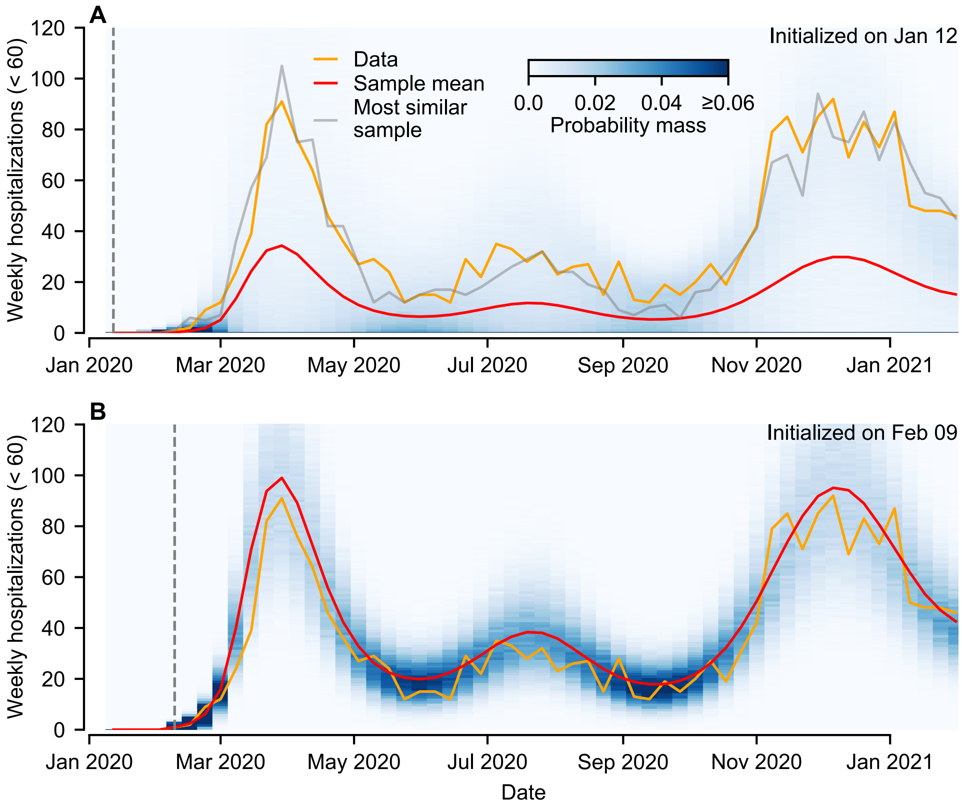

l 167 - While the stuff about Qt is interesting, it could be stressed more that this analysis only gives a vague, qualitative feel for the impact of stochasticity early in the pandemic, rather than a clearly interpretable parameter estimate or something (e.g., if there were a parameter in the model controlling the degree of heterogenity in transmission).

Q_t is a quantification of the impacts of stochastic events through time on the final week of data. A model parameter (e.g. an overdispersion parameter in a negative binomial model of transmission) would quantify the degree of heterogeneity overall but it wouldn't address the precise question we are interested in here: exactly when and to what extent did statistically atypical (low probability) transmission events occur.

An over-dispersed model of transmission changes the probabilities of those events, but it doesn't change the need for an analysis to quantify when they occurred (such as that shown in Figure 4D using Q_t).

In response to the reviewer's comments, we conducted simulations at the MLE using a negative binomial variant of the model. We have attached a repeat of Figure 5 using an overdispersion parameter $k = 0.3$ (where $k \to \infty$ corresponds to our original model without overdispersion). As might be expected, including overdispersion in the transmission process increased the variance across epidemic trajectories. However, our key qualitative finding—that atypically rapid early spread is necessary to explain the data—was unaffected.

l 212 - I wonder if the bit about early superspreading could be misinterpreted by readers. The model did not have any structure to handle superspreading per se, so this is really about stochasticity. The thing that I think is potentially misleading about how this is presented is that it could be interpreted to imply that superspreading is a general property of SARS-CoV-2 transmission that was also in effect later in the pandemic. While the reality is that that probably is the case, I do not see how this study provides support for an assertion like that. As such, I think the authors should be much more careful in how the present the conclusions drawn on this matter.

We appreciate the reviewer's concern that our original presentation may confuse readers. Our results show that, in order to be consistent with the data, the first few infections generated many more secondary cases than the mean of the offspring distribution. Given that these stochastic events fall in the tail of of the distribution, we feel justified in calling them specific instances of super-spreading (perhaps along the lines of isolated "super-spreading events"). However, what we did not do, which is more common in studies modelling super-spreading, is investigate whether X% of cases are responsible for Y% of overall transmission over the entire time period under study. To avoid possible confusion, we have changed the terminology to "atypically rapid early spread", to focus in on exactly what we are referring to.

l 217 - In this paragraphs, the authors seem to want to have it both ways. On the one hand, aggregating at the state level is too much. On the other hand, things were pretty synchronized and similar.

We appreciate how the reviewer arrived at this conclusion and recognise our original wording was not clear enough. What we mean is that there is a "baseline" grouping of counties which are geographically spread out across the state with a "remarkable degree of synchrony". However, this group (19 of 39 counties) excludes a number of counties with distinctive dynamics (including King and Snohomish counties, which together contain 40% of the state's population). Aggregating across the whole state would result in a misleading picture of epidemic spread. We have heavily reworked the discussion to make this clearer,

> "Our clustering analysis is able to identify a "typical" Washington state COVID-19 trajectory in 2020: three temporal waves of successively increasing magnitude in spring, summer and winter (the pink cluster shown in Figure 2). The presence of this "typical" highly-correlated cluster stands in contrast with the "travelling wave" pattern of spatial spread, observed for many human infectious diseases (including dengue [10], pertussis [44], measles [14], and influenza [15]). Instead, this cluster shows a remarkable degree of synchrony in the epidemic trajectories of counties from across the state. For instance, the mid-sized Clark, Spokane and Thurston counties are among the most highly rank correlated (see Figure 2A) and have a near-simultaneous initial epidemic take-off (see S10 Fig.) despite their geographic separation. The drivers of this synchrony are likely complex and

multifaceted, however we suspect it stems from a combination of i) near-simultaneous seeding of the epidemic across the state (aside from the early seeding of the Seattle metropolitan area) and ii) state-wide imposition of social distancing measures (including a prohibition of large gatherings and restaurant closures in spring and a mask mandate in summer 2020) [9]

"Importantly, we identify four clusters of contiguous counties that deviate from this typical trajectory. For instance, the yellow cluster, centered on King and Snohomish counties (together 40% of the state's population) had an atypically early and large initial wave, likely due to a combination of it being seeded directly from China [7, 8] and rapid early transmission (see below). We hypothesize that other factors were at play in the orange Yakima valley cluster, where the initial non-pharmaceutical interventions in spring 2020 appear to have been less effective at controlling transmission than elsewhere, prolonging the spring wave into the summer. Our clustering analysis highlights how state-level indicators can be misleading, for instance suggesting a growing state-wide epidemic when infections are confined to specific regions, or delaying the identification of transmission hot spots, such as in the Yakima valley during April 2020."

l 304 - during during

We thank the reviewer for catching this typo.

Reviewer #2: In the study, authors used two statistical methods to examine the spatial transmission pattern of SARS-COV-2 in Washington State during the 1st and 2nd wave (prior to mass vaccination/ VOCs). The first method (clustering analysis) identified several spatial clusters and the 2nd used a metapopulation spatial model along with the IF to estimate key parameters and infer underlying transmission dynamics for a subset of the WA counties at that time. The paper addresses important questions and reads interesting. However, it has some major errors/problems that require a reanalysis and likely revision of their main findings and conclusions.

Major issues:
1. The foremost problem with the paper is that, based on earlier case reporting/studies, authors assumed that SARS-COV-2 was introduced into WA in mid-January 2020 and continued to spread afterwards. This assumption was used to formulate the initial model conditions and substantially determined their model results and the conclusion that "super-spreading" has to occur to have the observed dynamics (i.e. little transmission prior to March 2020, followed by explosive expansion of infection). However, a later study in Worobey et al. 2020 Science (https://www.science.org/doi/10.1126/science.abc8169) showed that, while SARS-COV-2 was initially introduced in mid-Jan 2020, that initial spread was eliminated due to extensive public health intervention at

that time; the later pandemic wave was caused by subsequent reintroduction later on, rather than from the presumed silent, continued transmission since mid-Jan 2020. Because of this major factual error/assumption and its impact on model inference findings, authors should redo their 2nd analysis per findings from Worobey et al.

We thank the reviewer for bringing this highly relevant paper to our attention, which somehow evaded us. As the reviewer states, our model assumed that SARS-COV-2 was first introduced into WA mid-January 2020. However, we respectfully push back on the comment that this resulted in our model being erroneous. This is because our model included an importation term in the force of infection (with on average η introductions per week), hence our model did not require that all subsequent cases were descended from this initial case.

That said, our maximum-likelihood estimate was $\eta = 0$, which led us to conclude that the introduction in Snohomish county on January 12 2020 was likely to be the index case for the outbreak (in line with Bedford et al.). However, per the findings of Worobey et al., this case was unlikely to have seeded the resulting outbreak in the Puget sound region. Instead, they infer that a separate introduction on February 1 (with a 95% highest posterior density (HPD) of January 14 to February 15) seeded the resulting outbreak.

Following the reviewer's suggestion, we repeated the entirety of our inference assuming an introduction in the week of February 1. We find "that the maximum log-likelihood of the data post-January 26 are 7.73 log-likelihoods lower compared with using a start date of the week beginning January 12." In addition, the maximum likelihood estimate for η suggests an average of 8 introductions per week, making it extremely improbable that all cases in the outbreak were descended from a single introduction (the finding of Worobey et al).

In short, the results of our analysis of epidemiological data suggest that a single introduction on February 1 is unlikely to have generated a sufficiently large outbreak to account for the number of observed cases and hospitalisations in early March (after community transmission was detected but before the implementation of social distancing measures on March 16. Our results favour a single introduction as sparking the outbreak (not necessarily the January 15 case in Snohomish county), but suggest the epidemiological data favour an importation time at the earlier end of the 95% HPD of Worobey et al.

As we explain in the revised text, the "atypically rapid early spread" is a generic feature of stochastic dynamics where the outbreak successfully establishes itself from a single introduction. Indeed, the phenomenon is also observed when we repeat our analysis initialising the simulations in the week of February 1 with $\eta = 0$ (S11 Fig.).

We have revised our introduction to make clearer the state of scientific knowledge before our study,

> “The first confirmed SARS-CoV-2 infection in the US was on January 15 2020 in Snohomish county, Washington state [4], less than three weeks after its reported discovery in Wuhan, prompting intensive contact tracing efforts [5]. A lack of community testing meant that sustained transmission was not suspected until an outbreak in a long-term care facility was detected in neighbouring King county on February 28 [6]. Phylogenetic analysis suggests that cryptic community transmission had been ongoing in Washington state since late January, likely due to a separate introduction from China [7], and that cases were substantially under-reported [8].”

We have also amended the discussion,

> "Phylogenetic evidence suggests that, while the Puget sound outbreak was likely seeded from a single introduction, it was unlikely to stem from the initial introduction in Snohomish county [7]. Instead, Worobey et al. find that the outbreak was likely sparked by separate undetected introduction on February 1 2020 (with a 95% highest posterior density (HPD) of January 14 to February 15) [7]. Our results are not inconsistent with other introductions sparking the outbreak ($\eta \leq 2$ is within the 95% confidence interval, see Figure 3C). After repeating our analysis using an outbreak start date of the week beginning January 26 (S8 Fig. and S9 Fig.), we find that the maximum log-likelihood of the data post-January 26 is 7.73 log-likelihoods lower compared with using our original start date, the week beginning January 12. Furthermore, $\eta = 0$ is outside the 95% confidence interval, implying that the later start date requires a number of additional introductions in order for the epidemic to be large enough to be consistent with the hospitalizations data. In summary, while our results do not necessarily require the outbreak to be seeded by the Snohomish introduction, in order to explain the number of hospitalizations in March and April the epidemiological data favour the putative undetected introduction occurring at the beginning of the 95% HPD of Worobey et al.”

2. Authors used hospitalization data (for ages under 60) for model inference (2nd analysis). In the model, the parameter rho (probability of an infection is under 60 and hospitalized) was used to link model-estimated infection to the under 60 hospitalization data. Rho is a combination of two probabilities, both could change over time. First, the probability of an infection being under 60 could change and likely did change substantially over time due to changes in infection demographics, as different interventions affected different population segments over time. The 2nd probability, hospitalization given infection, also changed over time; particularly, with better case management and outpatient treatment, hospitalization rate decreased in later months (likely after the initial wave, but I don’t have a good reference for this; so please check the literature). So in combination, I would expect rho to vary, not so trivially, over time; and as such, treating rho as a constant parameter (even though it is estimated per the data) would be problematic.

We appreciate that that the number of hospitalised cases under 60 is not a perfect time invariant fraction of the number of total infections, but we do consider it a much better match than either confirmed cases (which were subject to well publicized testing issues early on) and mortality data (which are subject to changes in case outcomes and patient survival — see Refs. 53 and 54). Based on a preliminary analysis when we originally designed our study, which we now include as a supplementary figure, we do not believe the age distribution of infectious varied substantially in the Puget sound region over the course of 2020 (S6 Fig.). The ratio of hospitalised cases over 60 to those aged 20-69 gradually declined from March to May 2020, which we speculate was due to increased social distancing by older individuals who were most at risk. On these grounds, we feel justified in not performing our statistical inference using an explicitly age and spatially structured model, which would have been prohibitive on computational grounds.

To address the second part of the comment, we have added a new paragraph to the discussion,

> "Uncertainty in data quality has proven a challenge to understanding the early spread of the SARS-CoV-2 pandemic. As we explained above, we chose to perform our inference on time series data of hospitalised cases aged under 60 as they were unaffected by well established time-varying biases, in particular due to test availability issues and improvements in hospitalized patient outcomes [53, 54]. That said, hospitalisation data may be affected by time varying trends in care seeking behaviour (e.g. due to public awareness) or hospital admission criteria (e.g. due to hospital capacity limitations). Unfortunately, we were unable to find documented evidence of the extent and timing of these possible biases."

Looking at Fig 7, the estimated cumulative infection rates were fairly low. Authors overlapped their estimates with the CDC serology data from Sep 2020 onward to show their estimates were in line with the serology data. But serology data have issues due to sero-revision. So I wonder if the model underestimated the infection rate due to the use of the constant rho. At least some sensitivity analysis is needed to address this.

We have heavily revised Figure 7 by including independent estimates of the cumulative prevalence of SARS-CoV-2 infection from four other studies. We find that "our simulations are broadly consistent with two studies (Davis et al. and Wu et al.), and are smaller than the estimates of Pei et al. and Reese et al. by a factor about 1.5 and 3 respectively."

The issue of seroreversion is an important one, and we have added text commenting on it to the discussion,

> "Our reconstruction of the cumulative prevalence of infection in the Puget Sound region suggests that over the course of 2020 about 7% of the population were exposed to SARS-CoV-2. While our reconstructed prevalence is consistent with two previous studies [38, 41], it also broadly tracks the estimated seroprevalence

> in Washington state published by the CDC [42]. Given this estimate has not been corrected for seroreversion [43], it likely provides an underestimate of the overall percentage of the population exposed to SARS-CoV-2. Our reconstructed prevalence is also lower that in two other studies [39, 40]. This difference could in part be explained by our use of the MLE for model parameters in our reconstruction. In fitting the data, there is a trade-off in model performance between outbreak size and the parameter ρ, the probability an infection was aged under 60 and hospitalized: As ρ decreases a larger epidemic is needed to fit the data. The 95% confidence interval on ρ allows for epidemics that are about 50% larger, which would be broadly consistent with Pei et al., however the estimate of Reese et al., from a study using serological data, is about 300% larger [40]. This discrepancy may stem from their estimate being for the combined states of Alaska, Idaho, Oregon and Washington."

Other comments:
3. 1st paragraph of Introduction: i) SARS-COV-2 was detected in Dec 2019, not Jan 2020;

While SARS-CoV-2 has been detected in samples from December 2019, the virus was not "discovered" until January 2020, with the first public announcement of the discovery of a novel coronavirus on January 7th 2020. Although our original statement was factually correct, we have changed it to "detected in samples from Wuhan, China from December 2019", to avoid possible reader confusion.

ii) problem with the description of the initial introduction, as noted in comment #1;

We have amended the introduction to explain that "Phylogenetic analysis suggests that cryptic community transmission had been ongoing in Washington state since late January, likely due to a separate introduction from China [7],

iii) omega should be Omicron.

We thank the reviewer for spotting this mistake.

4. Analysis 1. Only case time series were used for the clustering analysis. Have authors tried using hospitalization and mortality data as well? And how consistent are the results, given all those data have some issues/biases.

We repeated the analysis on weekly hospitalizations data, which we have included as a new supplemental figure. We found that

> "The data were substantially noisier, due to the smaller numbers of infections that were hospitalized -- reflected in lower entries in the correlation matrix. Overall,

the clusters were less well defined, as quantified using the silhouette score (S5 Fig.). As might be expected, the impact of increased noise is most pronounced on the clustering of smaller counties. Apart from Thurston, the top ten most populous counties all remain in the same clusters (S4 Fig.)."

5. Page 7, the very large drop in R_i^res in Thurston and Kitsap. Looking at Fig 4, there were much fewer hospitalizations in those counties. How much is this estimate due to the much lower level of early transmission in those counties, rather than a drop due to lockdown?

This estimate is determined by the lower levels of transmission in these counties, i.e. to be consistent with the data. If, all other factors the same, R_i^{res} didn't drop by the amount it did then the model would predict the epidemic grew faster and become larger in those two counties than it did.

In principle, prior to the lockdown the initial reproductive number could have been lower in these two counties, however the inclusion of additional model parameters would result in identifiability issues. We therefore make the parsimonious assumption that the reproductive numbers were the same prior to lockdown.

We suspect that the rapid drop in R_i^{res} in these two counties might be related to the changes in mobility, as we comment on in the revised discussion,

> "Our analysis highlights some of the challenges in constraining spatial epidemiological models using census commuting data, a common practice in epidemiological modeling (see e.g. [49–52]). Firstly, the rapid spread of SARS-CoV-2 from Snohomish to King county may have been facilitated the mobility patterns of certain key populations groupings, for instance healthcare workers, who are not representative of the population average captured in the census data. Secondly, while for most of 2020 we observed that model performance was positively correlated with connectivity strength, during the period of most intensive social distancing (April – June), this correlation was inverted. Instead, performance was better at smaller values of the commuting parameter. If non-pharmaceutical interventions resulted in a disproportionately large reduction in inter-county transmission compared to intra-county transmission, as was likely the case, this would explain the reversal in model performance. This might also explain the large drops in R_i^{res} observed in Kitsap and Thurston counties during this period, the two counties with the lowest per capita hospitalizations. Parameterising models using accurate time-varying data on human mobility would help alleviate this issue. Overall, however, our results show this second phenomenon was of smaller importance to model performance than the early rapid transmission outlined above."

6. Page 9 and Fig 5. I don't understand what the figure is showing, especially the results on Qt and Fig 5D. Is Qt cumulative or for each week? If Qt is cumulative, it does not look like there is much difference after March 2020. Could authors please clarify?

We have amended the text to more clearly introduce Q_t, namely that it is the

> "the log-probability of the final week's data (week beginning January 31 2021), x_T, conditioned on all data up-to-and-including week t, $Q_t = \ln P(x_T | \{x_s\}_{s \leq t})$. An increase (decrease) in Q_t in week t implies that the new observation x_t increases (decreases) the probability of observing x_T at the later time T."

While in each week Q_t involves conditioning on incrementally more data, it is not a cumulative probability distribution and it is not cumulative in the sense that Q_t can be expressed as a sum involving Q_{t-1}.

The reviewer is absolutely correct in observing that it doesn't increase much after March 2020, this is our main take-away from the figure,

> "We see the largest rise in Q_t during March and April, implying that the impact of early stochastic events persisted over the entire period of study. Subsequently, there is little increase in Q_t between April and October 2020, implying that individual stochastic fluctuations have limited lasting impact."

7. Hospitalization data have issues that warrant some discussion on study limitation. Before March 2020, there was limited testing, leading to under counting. It could also be affected by healthcare seeking behavior, e.g., awareness and concern of covid could drive more people to seek treatment. These systemic biases/similar behavior across population that affected the data could have contributed to the apparent synchrony.

We have added paragraph in the discussion on these potential data quality issues, see our response to comment 2.

Reviewer #3: This is quite an interesting paper looking at whether / why / how we can reconstruct effective connectivity matrices between spatial regions according to the reported case counts at that scale. I think the paper is quite well written, the figures are clear, and the approach itself is interesting. While I have a tough time imagining this technique being deployed for nearly real-time policy response (it would seem to require more data to validate if one were applying this technique in real-time), it can be used to retrospectively characterize the often hidden connectivity between regions. I think that'a great! There are a couple small things I have questions about, but overall, I think this is a really nice paper that doesn't require too many tweaks to see it through.

We are pleased to see the positive impression the reviewer has of our work.

*1. I have a few broad questions about robustness of these measures to spatial/temporal up/down-scaling, which I think could improve this method's usefulness in broader contexts outside of Washington. The reason for this question is that I'm imagining what it would look like to apply it in other contexts, say, Arizona and Massachusetts (I'm not asking for this exact analysis, just as examples). Both of these states have ~15 counties, but Massachusetts reports much of its case counts / hospitalization data at the *municipality* level in addition to counties (n=351 vs n=15). Municipalities in MA are largely subsumed by counties, so in theory, one could start from a correlation matrix as in Fig 2A for both the county-level and municipality-level correlations. The reason I'm curious about these different spatial scales is because of the authors' discussion and analyses relating to super-spreading in the early months of the pandemic. The authors' explanation is largely compelling, but would that same conclusion be drawn if only we had more spatially granular data of case counts?*

The reviewer makes an important comment on the relevance of spatial scale. Our conclusions about atypically rapid early transmission being responsible for the establishment of a successful introduction is a generic result that stems from the properties of stochastic birth-death processes, which we have commented on in the revised discussion

> "In addition to requiring high spatial connectivity, we also found the outbreaks in both King and Snohomish counties at the start of lockdown were roughly twice and three times larger respectively than would be expected from a typical (mean) realization of the best-fitting model (see Figure 6). If the outbreak was seeded from a single later introduction in the week of January 26 (see previous paragraph), the same result occurs (S11 Fig.). This observed phenomenon is due to demographic stochasticity in the disease transmission process, and suggests that atypically large bursts of transmission occurred in the first couple of weeks. Dynamically, this is a property of stochastic birth-death processes [55], of which the early phase of an outbreak is an example. In essence, an outbreak sparked in a population that successfully avoids dying out is more likely to have spread faster than average — mathematically, if the probability of (local) extinction is $ $P(X_t = 0) > 0$ then $\mathbb{E}[X_t | X_t > 0] > \mathbb{E}[X_t]$. The impact of individual high transmission events on avoiding extinction is more pronounced the earlier in the outbreak they occur, and their occurrence has the effect of ``fast-fowarding'' the epidemic, with the resulting epidemic size being larger than would be expected from the mean trajectory."

We therefore don't expect these findings to be impacted by the granularity of the data, as they are generic properties of the transmission process. We also expect that, with the appropriate parameterisation, the overall model performance would improve as the

granularity of the case counts data increases. The key limitation, however, would be the spatial granularity of the mobility data. In our inference analysis the model was parameterised using census commuting data, which is only available on the county level.

More generally, it would be helpful to know how the results depend (or don't) on the selection of spatial scale that cases are reported at. One approach would be to artificially merge Washington counties into "double counties" and re-run the analyses as if Washington had half the number of counties but ones that were larger in size and population than they currently are. You can do this again and again until all the counties form a single mega-county that subsumes the whole state --- in that case, the reported cases time series at the state level would be perfectly synchronized (trivially so, as it is only being compared with itself), whereas one can imagine the spatial correlation between cases at the arbitrarily small scale (e.g. partitioning the state of Washington into tons of 20x20 meter parcels) may not be as informative as the scale used in this paper.

I would like to see the authors contend with this a bit more, addressing and attempting to quantify how exactly the effectiveness of this approach depends on the spatial scale of the reported data and the temporal scale at which it is reported (i.e. the same discussion above is still relevant when imagining up-sampled or down-sampled time series data; how would this approach change if data were only reported at 2-week intervals? 1-week? Daily?).

The data were made available at the weekly resolution. Performing the clustering analysis on cases aggregated by month results in three clusters being favoured by the silhouette score: one combining the yellow and pink, one combining orange and green, and the original blue cluster. The sole difference is that Ferry county (which sparsely populated and the most distantly connected in the original analysis) attaches instead to the yellow + pink cluster.

Broadly, we do expect the results to depend on spatial scale. As the reviewer suggests, at extremely fine scales there would be a general reduction in spatial correlation and an increase in the noisiness of each $\rho_{i,j}$ (something we also witness when we calculate the correlation matrix using hospitalisations data — effectively a subsampling of the case data, see S4 Fig.). We find that,

> “Overall, the clusters were less well defined, as quantified using the silhouette score (S5 Fig.). As might be expected, the impact of increased noise is most pronounced on the clustering of smaller counties. Apart from Thurston, the top ten most populous counties all remain in the same clusters (S4 Fig.).”

We weren’t able to think of a way to systematically progressively merge counties, re-run the clustering analysis and then interpret the results. Broadly we expect the effect of

merging counties to depend on if they were in the same cluster originally, and also the population size of the counties (a proxy for how noisy the time series are).

As a test we trialed a couple of “double county” mergers. For instance we found that merging Pierce county and King county (the two largest counties, originally pink and yellow clusters, respectively) resulted in the combined county belonging to the yellow cluster (King county’s cluster) with the rest of the clustering unaffected. Merging Chelan and Douglas county (both originally green) resulted in the green and orange clusters combining. Merging Yakima and Franklin (both originally orange) resulted in the Cowlitz, Kittitas, Skamania and Whatcom grouping attaching to the orange county rather the pink. This grouping (aside from Whatcom) becomes contiguous with the enlarged orange cluster and is the most distantly connected grouping not identified as a distinct cluster (as based on the silhouette score).

These tentative results suggest that merging counties (i.e. increasing spatial scale) can alter the exact clustering, especially the merging of smaller rural counties that are more weakly correlated. However, the overall geographic patterning of the clustering remains robust.

2. Stepping back, it also seems like the authors could use this to provide stronger / more normative explanations about data reporting procedures nationwide. That is, if this approach can prove to be a useful stand-in or complement to the large scale mobility datasets that some research groups have access to, what is the best / most appropriate spatial scale to report data at to ensure that the spatial granularity is maximally informative to the virus that's spreading?

We certainly agree with the reviewer. Our results demonstrate how epidemiological data can be used to constrain uncertain mobility parameters. We view inference approaches such as ours as best serving as a complement to reliable large mobility datasets (such as the commuting data used in our analysis), with the mobility datasets helping to constrain the model parameterisation. In the absence of any mobility data inferential approaches such as ours can be used to reconstruct mobility matrices, however they will quickly run into both computational and identifiability issues as the number of distinct spatial locations grows.

In some ways we view our analysis as providing an upper-bound on the scale necessary to understanding the spatial spread of SARS-CoV-2, with the state (let-alone country) level being inadequate. When and where there is a similar importance of even finer spatial scales in determining the spread of SARS-CoV-2 is not something we can answer with the data we had available (which were provided on the county level).

Broadly we would say that reporting of epidemiological data is best at the finest scale possible (e.g. census block or zip code), provided mobility parameters at the same scale can be reliably obtained. That said, inference using such fine-scale models would

likely become computationally prohibitive for modelling spatial regions larger than a single metropolitan area.

3. Lastly, I really think the idea of a "resident reproductive number", and I think its use here is important for future work looking at the potentially vast spatial heterogeneities that may exist depending on whether the R_i^{res} *is calculated at the census tract level, county, state, etc.*

We appreciate the reviewer's thoughts of the resident reproductive number. In a separate study we have found it particularly useful when studying the nationwide spread of SARS-CoV-2 on the county-level. In essence, using the standard next-generation method to calculate R_0 roughly translates into finding the county with the largest R_i^{res} and provides very limited insight into the underlying dynamics.

4. Very small: add a "by" to line 275 -- "may have been facilitated ___ the mobility patterns"

We thank the reviewer for catching this typo.
