# Supplementary material for: Charting the spatial dynamics of early SARS-CoV-2 transmission in Washington state
Source: PLoS Comput Biol. 2023 Jun 28;19(6):e1011263. doi: 10.1371/journal.pcbi.1011263 (PMC10335681; doi:10.1371/journal.pcbi.1011263)
Supplement: Attachment — Submitted filename: COVID_19_clustering_second_response_to_reviewers_2023_05_26.pdf [file pcbi.1011263.s013.pdf]

Response to reviewers' comments

Comments to the Authors:
Please note here if the review is uploaded as an attachment.

Reviewer #1: I am satisfied with the authors' extensive revisions.

Reviewer #2: I appreciate the authors revise the paper to address my comments. There are a few things I still don't agree/understand:

1) Worobey et al. showed that there was at least one additional introduction in early February that likely led to the subsequent pandemic wave in WA. I don't think they precluded additional introductions or multiple introductions around that time. And given that SARS-COV-2 had expanded globally by Feb/March 2020, with multiple outbreaks in Europe and silent spread in places like NYC, multiple introductions around Feb 2020 was more likely than not. So I don't understand the authors' argument that b/c eta was estimated to be ~8, it was impossible to have a later start of the pandemic wave in Feb 2020.

We fear the reviewer may have misunderstood our paper — at no point do we state that it is impossible for the epidemic to have been sparked by more than one introduction — as we state in the discussion, "our results are not inconsistent with other introductions sparking the outbreak ($\eta \leq 2$ is within the 95% confidence interval, see Figure 3C)." Instead, based on two lines of reasoning informed by our analysis of the epidemiological data, we suggest a later introduction date is disfavoured. Firstly, regardless of the phylogenetic data, the epidemiological data disfavour a later start date:

> "After repeating our analysis using an outbreak start date of the week beginning January 26 (S8 Fig. and S9 Fig.), we find that the maximum log-likelihood of the data post-January 26 is 7.73 log-likelihoods lower compared with using our original start date, the week beginning January 12… In summary, while our results do not necessarily require the outbreak to be seeded by the Snohomish introduction, in order to explain the number of hospitalizations in March and April the epidemiological data favour a putative undetected introduction occurring at the beginning of the 95% HPD of Worobey et al."

The second line of reasoning for discounting a later start date is based on the fact that the results of Worobey et al. suggest that the WA outbreak clade descends from a single introduction, whereas our MLE using the later start date has an average of 8 weekly introductions (an MLE which we stress is 7.7 log-likelihoods lower compared with using our original start date — see above). These two estimates are unlikely to be reconcilable, which we take to be further support for the outbreak being seeded by "a putative undetected introduction occurring at the beginning of the 95% HPD of Worobey et al."

The hypothetical alternative scenarios the reviewer lists are unlikely to be plausible on phylogenetic grounds. As Worobey et al. state,

> "The posterior maximum clade credibility (MCC) tree (Fig. 4) suggests that the WA outbreak clade (plus S566 and a sibling virus sampled in New York, "NY") resulted from an introduction from Zhejiang, China, as supported by the clustering of sampled and unsampled taxa from this location. Additionally, although an introduction from a Chinese location other than Hubei yields considerable posterior support (bar chart inset in Fig. 4), Hubei is preferred over Zhejiang for the entire posterior sample as the most likely source for this introduction. "

The WA outbreak clade is extremely unlikely to be derived from introductions from Europe or NYC as they fall in a separate branch of the tree (see Figure 5 of Worobey et al). Therefore these locations can be discounted as seeds for the outbreak.

If there were multiple introductions into Washington from China that seeded the outbreak (i.e. a WA3, WA4, …) then the results of Worobey et al. suggest they would have to share a most recent common ancestor (MRCA) around the end of January (see their Figure 4). However, at that time the size of the outbreak in China was likely in the 1000s of cases. Therefore every additional introduction from China substantially decreases the probability that all the observed cases in WA share a common ancestor at the end of January. ***In summary, although the phylogenetic evidence does not formally exclude it, an average of 8 introductions per week starting from February 1 makes the results of Worobey extremely improbable.*** This is the reasoning that motivated Worobey et al. to write about a single unobserved introduction (WA2, see Figure 6) rather than a (comparatively large) number of unobserved introductions that each sparked sustained chains of transmission.

To make this point clearer, and to better match the exact text of Worobey et al., we have made a small change to the relevant sentence in the results section, from "[our results make] it extremely improbable that all infections in the outbreak were descended from a single introduction (the finding of Worobey et al. [7])" to "[our results make] it extremely improbable that the WA outbreak clade resulted from a single introduction (as suggested by the results of Worobey et al. [7])".

Relatedly, eta (the seeding) is probably time-varying. So it's probably not appropriate to treat it as a constant. Similar problems exist for other time-varying parameters (rho etc.). So in general, I think it is somewhat problematic to use the IF, which is designed for time-stationary parameter estimates, for the study here. At the least, I'd hope the authors use larger perturbations in the IF to add the necessary uncertainty to those time-varying parameters.

As always with epidemiological modelling, simplifications need to be made. While it is obviously true that the importation rate changed in time, we have no way of reliably constraining this variation. The impact of the introduction rate on transmission (and

therefore the log-likelihood) is largest early on when the number of infections is low (as can be seen by inspecting the force of infection, Eq. 3), and shrinks rapidly as the epidemic grows exponentially. We therefore reason that for the goal of our study, which is to understand the spatial dynamics of SARS-CoV-2 in WA, assuming a constant introduction rate is justified.

It is incorrect to assert that iterated filtering is not suited to estimating time-varying parameters. This is accomplished in iterated filtering by augmenting the parameter space with a parameter set that parameterises an appropriate time-varying function. Indeed, this is how we estimate the local reproductive number in each county, which is parameterised by estimating the coefficients for a set of basis splines (see Eqs. 6 and 7). As we state above, additional time-varying parameters would substantially enlarge the search space, introducing potential identifiability complications without providing much additional gain in performance.

Time-varying trends in the reporting probability, ρ, are something we discuss in the manuscript, see for instance this paragraph from the discussion,

> "As we explained above, we chose to perform our inference on time series data of hospitalised cases aged under 60 as they were unaffected by well established time-varying biases, in particular due to test availability issues and improvements in hospitalized patient outcomes [53, 54]. That said, hospitalisation data may be affected by time varying trends in care seeking behaviour (e.g. due to public awareness) or hospital admission criteria (e.g. due to hospital capacity limitations). Unfortunately, we were unable to find documented evidence of the extent and timing of these possible biases."

2) I don't understand what Fig S6 is showing nor the authors' reply to my comment related to the changing hospitalization-to-infection ratio. The data show hospitalizations (i.e. the nominator) yet the denominator –i.e., infections – is not observed and infection demographics likely changed substantially, due to changing NPIs and mobility. Unless the model is age-specific and uses age-specific hospitalization-to-infection ratio, the non-age-specific hospitalization ratio likely would vary with the infection demographics and it probably would not be so trivial.

Our reasoning can be understood as follows. To a first approximation, (i.e ignoring demographic stochasticity and hospitalisation delays, which don't impact the argument, just make the notation more complicated) the number of hospitalisations in age class *i* at time *t*, $H_i(t)$, is given by

$$H_i(t) = \rho_i(t)I_i(t),$$

where $\rho_i(t)$ is the age-specific hospitalisation probability and $I_i(t)$ is the number of new infections in age class i.

If we consider the ratio for two different age classes (which is the quantity plotted in Fig S6D), then

$$\frac{H_1(t)}{H_2(t)} = \frac{\rho_1(t)I_1(t)}{\rho_2(t)I_2(t)} = \frac{\rho_1(t)}{\rho_2(t)}\frac{I_1(t)}{I_2(t)}.$$

If the ratio $H_1(t)/H_2(t)$ is approximately constant (which is what we observe from the start of March onwards for all age-classes under 60) then either: i) the ratio of hospitalisation probabilities $\rho_1(t)/\rho_2(t)$ and the ratio of infections $I_1(t)/I_2(t)$ are both roughly constant or ii) the both change in such a way that the changes close to cancel out. We consider the first explanation more plausible, especially given the lack of evidence on substantial variation in $\rho_i(t)$ (e.g. due to changes in disease severity). Notice that the same phenomenon is observed in confirmed cases after June 2020, likely due to the resolution of testing supply issues, which lends further support to the first explanation. Together, this evidence presented in Figure S6 suggests that while there may have been social distancing measures that targeted specific age-groups, and that they had an impact on overall incidence, they did not substantially change the age profile of infections under 60.

This reasoning then informs the construction of the model used in our study, and why an explicitly age-structured model is unnecessary. The total number of weekly hospitalisations under 60 is then given by

$$H(t) = \sum_i \rho_i(t)I_i(t),$$

where the sum runs over all age groups under 60. We assume the age-profile of infections for age-groups under 60 is approximately constant, $f_i = \frac{I_i(t)}{\sum_i I_i(t)}$. The number of hospitalisations under 60 is then,

$$H(t) = \left(\sum_i \rho_i(t)f_i\right)I(t),$$

where $I(t) = \sum_i I_i(t)$. Assuming that $\rho_i(t)$ doesn't change much over the period of study (see above) we then have

$$H(t) = \rho I(t),$$

with $\rho = \sum_i \rho_i(t)f_i$ being the average probability of hospitalisation for under 60 y. o. infections, the parameter that is estimated in the main paper.

Our modelling therefore relies on the assumption that the hospitalisation probably for individuals under 60 does not vary substantially over the period of study. While we consider this assumption to be supported by evidence, we do comment in the discussion,

> “Uncertainty in data quality has proven a challenge to understanding the early spread of the SARS-CoV-2 pandemic. As we explained above, we chose to perform our inference on time series data of hospitalised cases aged under 60 as they were unaffected by well established time-varying biases, in particular due to test availability issues and improvements in hospitalized patient outcomes [53, 54]. That said, hospitalisation data may be affected by time varying trends in care seeking behaviour (e.g. due to public awareness) or hospital admission criteria (e.g. due to hospital capacity limitations). Unfortunately, we were unable to find documented evidence of the extent and timing of these possible biases.”

3) Authors commented on hospitalization data being too noisy for their 1st analysis (clustering analysis) yet used hospitalization data for the 2nd analysis. That sounds contradictory.

We recognise at first glance this might appear contradictory, however this is not the case. The reason noisiness in hospitalisation data is not an issue for the second analysis is twofold: 1) the analysis focuses on a subset of counties with large populations, 2) the analysis models the data using a partially-observed Markov process results in greater robustness to incomplete and noisy data.

In particular, all of the five counties used in the second analysis are among the top ten largest counties by population size — with Kitsap county being the smallest (population of 274,314 individuals). In contrast, the smallest county used in the clustering analysis was Ferry county, with a population of 7,448 individuals. When we repeated the clustering analysis using hospitalisation data (see SFig. 4), the epidemic trajectory in these small counties is lost to noise. For larger counties the signal of the epidemic trajectory is more robust when using hospitalisation data, and, as we state in the manuscript, “[a]part from Thurston, the top ten most populous counties all remain in the same clusters”.

4) “We see the largest rise in Qt during March and April, implying that the impact of early stochastic events persisted over the entire period of study. Subsequently, there is little increase in Qt between April and October 2020, implying that individual stochastic fluctuations have limited lasting impact.” I don’t understand this take. If there was not much change in Qt after April 2020, “implying that individual stochastic fluctuations have limited lasting impact” (2nd part of the sentence), why the first part says “the impact of early stochastic events persisted over the entire period of study”?

We thank the reviewer for bringing this to our attention — our wording was ambiguous. We are referring to individual stochastic fluctuations which occurred during the period

April to October 2020 having limited lasting impact. This has been clarified in our new submission:

"We see the largest rise in Q_t during March and April, implying that the impact of early stochastic events persisted over the entire period of study. Subsequently, there is little increase in Q_t between April and October 2020, implying that individual stochastic fluctuations *which occurred during this second period* had limited lasting impact."

5) The closing sentence says "Finally, our results demonstrate that accurate prediction of future spread within densely populated urban areas during periods of low incidence is not feasible without: i) modelling approaches capable of accounting for stochastic transmission events and ii) access to zip-code- or neighbourhood-level epidemiological and mobility data, at a minimum." This is somewhat contradicting. If it is mostly stochastic, I don't think it is predictable, no?

Predictive models commonly incorporate stochasticity e.g. in finance, climate modelling etc. For an example of the use of stochastic models in predicting infectious disease dynamics see Viboud et al, 2018 (https://www.sciencedirect.com/science/article/pii/S1755436517301275).

Including stochasticity in dynamical models can, perhaps counterintuitively, make them more accurate by avoiding model misspecification — i.e. they better capture the modelled systems's dynamics, see e.g. King et al, 2015 (https://royalsocietypublishing.org/doi/10.1098/rspb.2015.0347). For instance, one particular issue with deterministic models is that the are unable to capture localised disease "fade-out" events, see Roberts et al, 2018 (https://www.ncbi.nlm.nih.gov/pmc/articles/PMC4996659/**)**. While the predictions of a deterministic model might have higher precision than a stochastic model (i.e. narrower uncertainty), by neglecting stochasticity in the dynamics they may have low accuracy (i.e. they can be confidently wrong).

For these reasons we stand by our original sentence, but we have reordered it to better emphasise that our two recommendations are the "minimum" requirements for "accurate prediction of future spread within densely populated urban areas", and therefore may not be sufficient.

6) Overall, I think there is substantial uncertainty (e.g., noted in my comments above) and simplification (some noted by the authors themselves)/likely misspecification in the study. Given the uncertainty, I would recommend the authors at least tone down their statements on the findings (e.g., sentences in the concluding paragraph).

We have gone over the manuscript and made minor improvements to the wording in a number of places to ensure readers do not misunderstand the scope of our findings.

Reviewer #3: The authors have conducted quite an extensive, impressive series of edits, which have satisfied my original questions and concerns—especially those related to the most-appropriate spatial scale for data in this procedure. I'd like to commend their efforts and note that I am satisfied with their revision.
